# Nerve Enlargement in Patients with INF2 Variants Causing Peripheral Neuropathy and Focal Segmental Glomerulosclerosis

**DOI:** 10.3390/biomedicines13010127

**Published:** 2025-01-08

**Authors:** Quynh Tran Thuy Huong, Linh Tran Nguyen Truc, Hiroko Ueda, Kenji Fukui, Koichiro Higasa, Yoshinori Sato, Shinichi Takeda, Motoshi Hattori, Hiroyasu Tsukaguchi

**Affiliations:** 1 Second Department of Internal Medicine, Division of Nephrology, Kansai Medical University, Hirakata 573-1010, Japan; 2 Department of Internal Medicine, Pham Ngoc Thach University of Medicine, Ho Chi Minh 70000, Vietnam; 3 Department of Biochemistry, Faculty of Medicine, Osaka Medical and Pharmaceutical University, Takatsuki 569-8686, Japan; 4 Department of Genome Analysis, Institute of Biomedical Science, Kansai Medical University, Hirakata 573-1010, Japan; 5 Department of Medicine, Division of Nephrology, Showa University School of Medicine, Fujigaoka Hospital, Yokohama 227-8501, Japan; 6 Internal Medicine, Kurobe City Hospital, Toyama 938-8502, Japan; 7 Department of Pediatric Nephrology, Tokyo Women’s Medical University, Tokyo 162-8666, Japan; 8 Clinical Genetics Center, Kansai Medical University Hospital, Hirakata 573-1010, Japan

**Keywords:** FSGS, Charcot–Marie–Tooth, schwannomatosis, formin, actin cytoskeleton

## Abstract

**Background**: Charcot–Marie–Tooth (CMT) disease is an inherited peripheral neuropathy primarily involving motor and sensory neurons. Mutations in INF2, an actin assembly factor, cause two diseases: peripheral neuropathy CMT-DIE (MIM614455) and/or focal segmental glomerulosclerosis (FSGS). These two phenotypes arise from the progressive degeneration affecting podocytes and Schwann cells. In general, nerve enlargement has been reported in 25% of the demyelinating CMT subtype (CMT1), while little is known about the CMT-DIE caused by INF2 variants. **Methods**: To characterize the peripheral nerve phenotype of INF2-related CMT, we studied the clinical course, imaging, histology, and germline genetic variants in two unrelated CMT-DIE patients. **Results**: Patient 1 (INF2 p.Gly73Asp) and patient 2 (p.Val108Asp) first noticed walking difficulties at 10 to 12 years old. Both of them were electrophysiologically diagnosed with demyelinating neuropathy. In patient 2, the sural nerve biopsy revealed an onion bulb formation. Both patients developed nephrotic syndrome almost simultaneously with CMT and progressed into renal failure at the age of 16 to 17 years. Around the age of 30 years, both patients manifested multiple hypertrophy of the trunk, plexus, and root in the cervical, brachial, lumbosacral nerves, and cauda equina. The histology of the cervical mass in patient 2 revealed Schwannoma. Exome analysis showed that patient 2 harbors a germline *LZTR1* p.Arg68Gly variant, while patient 1 has no schwannomatosis-related mutations. **Conclusions**: Peripheral neuropathy caused by INF2 variants may lead to the development of multifocal hypertrophy with age, likely due to the initial demyelination and subsequent Schwann cell proliferation. Schwannoma could co-occur when the tissues attain additional hits in schwannomatosis-related genes (e.g., *LZTR1*).

## 1. Introduction

CMT is an inherited peripheral neuropathy affecting motor and sensory neurons, with prevalence of 1 in 2500 [1]. Genetically, more than 90 genes have been linked to CMT, most of which affect fundamental cellular pathways, such as the mitochondrial function, cytoskeleton dynamics, and organelle trafficking pathways [2]. CMT is clinically classified into three subtypes based on electrophysiological criteria: a demyelinating form (CMT1), with a nerve conduction velocity (NCV) of <35 m/s; an axonal neuropathy form (CMT2), with an NCV of >45 m/s; and an intermediate form, with an NCV of 35–45 m/s [3]. With accumulating knowledge of the causative genes, the categories of the CMT subtypes have been refined based on molecular and genetic information.

CMT rarely co-occurs with the renal disorder of FSGS: a subtype, CMT-dominant intermediate (CMT-DIE, MIM 614455), is caused by mutations in the inverted formin-2 gene (*INF2*), encoding an actin assembly factor [4]. Such patients with dual CMT and FSGS phenotypes (CMT–FSGS) usually experience both renal and neuronal disorders almost simultaneously in the first or second decade of life. CMT–FSGS phenotypes are characterized by an intermediate nerve conduction (NC) disturbance with distal muscle weakness and steroid-resistant nephrotic syndrome (SRNS). Kidney disease progresses rapidly into end-stage renal disease (ESRD) by the age of 20 years. One-third of CMT-DIE families (4 out of 12) have sensorineural hearing disability [4]. INF2 is expressed preferentially in Schwann cells and podocytes and functionally regulates both actin polymerization and depolymerization [5,6,7]. INF2 comprises two major domains: the N-terminal regulatory domain (diaphanous inhibitory domain, DID) and the C-terminal actin assembly unit (diaphanous autoinhibitory domain, DAD). Actin processing activity is regulated by autoinhibition, which is mediated through self-intramolecular interaction between DID and DAD. *INF2* mutations causing the dual CMT–FSGS diseases exclusively cluster within residues 57 to 184 of the DID domain, which likely impedes the intramolecular binding DID–DAD interaction [4,5,6,8].

Previous studies indicate that 25% of the demyelinating CMT1 subtype, the most prevalent form of CMT caused by *PMP22* duplication, manifests as peripheral nerve enlargement [9,10]. Such hypertrophic neuropathy may be clinically recognized as palpable thickened nerves, particularly in the neck, or visible on imaging studies and could cause compression radiculopathy or myelopathy [9,10]. Nerve enlargements manifest with age, likely reflecting the repeated demyelination and subsequent Schwann cell proliferation [3,9]. Rare cases of CMT with concurrent nerve sheath tumors, e.g., neurofibroma (NF) or Schwannoma, have been reported [9,11,12]. CMT and NF could coincidentally coexist, given that the disease prevalence is around 1 in 2500~3500 individuals [13]. Alternatively, the two conditions may develop based on commonly shared mechanisms, indicating a possible convergence of their pathogenic pathways.

We here report new aspects of peripheral neuropathy in two unrelated CMT-DIE patients caused by INF2 variants and discuss the underlying mechanisms. First, we demonstrate for the first time, to the best of our knowledge, the imaging, histology, and time course of multifocal nerve hypertrophy in CMT-DIE patients, which affected the trunk as well as the roots in the cervical and lumbosacral nerves after the age of 30 years. Second, we show that the germline schwannomatosis-related LZTR1 variants may predispose the patients to hypertrophic changes. Our observations alert the clinician that nerve enlargement might be an unrecognized, late-onset complication of the INF2-related CMT-DIE. The nerve enlargement might pose a risk of tumorigenesis when the tissues attain the second/third hits of modifier genes.

## 2. Materials and Methods

### 2.1. Mutational Analysis

We chose two patients (1 and 2) out of four CMT–FSGS cases previously reported [14]. The reason is that the two had undergone a detailed neurological assessment, while the others had not yet been thoroughly evaluated. This study was approved by the Kansai Medical University institutional ethics committee, following the standards of the Human Study of Ministers Joint and the Declaration of Helsinki. Informed consent was obtained from all subjects. Genomic DNA was isolated from peripheral blood cells using a QIAamp DNA Blood isolation kit (QIAGEN, Venlo, The Netherlands). Whole-exome sequencing was conducted using an Illumina NovaSeq 6000 sequencer (Illumina Inc., San Diego, CA, USA) with a Twist Human Core Exome 2.0 kit (Twist Bioscience, San Francisco, CA, USA). After aligning the sequence reads onto the reference genome (GRCh38) using the Burrows–Wheeler aligner, downstream analyses, including marking duplicates, base quality recalibration, haplotype calling, joint variant calling, and variant quality score recalibration (VQSR), were processed using Picard and GATK version 3.8 according to the GATK Best Practice recommendations [15,16]. We examined all rare (a minor allele frequency of less than 1% in public databases) and nonsynonymous coding variants. Variants were confirmed by Sanger sequencing on both strands.

Regarding the pathogenicity of the variants, mutational effects were predicted in silico by several algorithms, including the PolyPhen2, SIFT, M-CAP, LRT, and Mutation Taster. A systematic, tiered approach was applied to analyze and interpret sequence data. Called variants were interpreted and classified according to the American College of Medical Genetics (ACMG) criteria [17]. Variants classified as pathogenic, likely pathogenic, or variant of unknown significance (category 1–3, according to ACMG) were further analyzed.

### 2.2. Structural Analysis

Structural modeling was performed using AlphaFold3 through the use of the amino acid sequence of human LZTR1 (UniProt Q8N653) and GTP-binding protein RIT1 (Uniprot Q92963) isoform 3 [18].

## 3. Results

**Figure 1:**
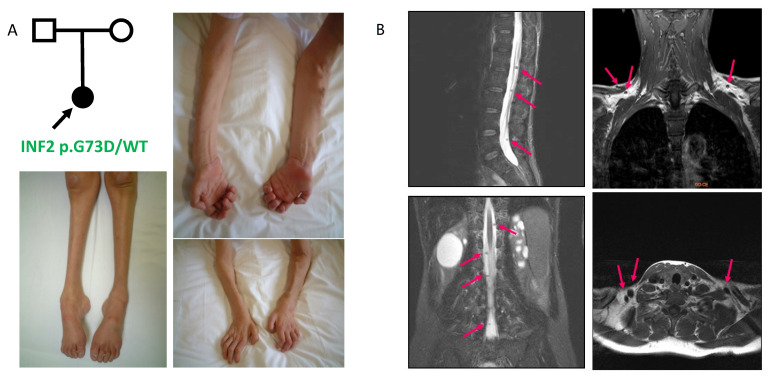
Clinical features of CMT–FSGS patient 1 with *INF2* p.G73D variant. (**A**). Pedigree and limb appearance. Pronounced muscle atrophy in her upper and lower limbs is seen at the age of 44 years. The distal muscles in the lower limb show typical appearance of pes cavus and clawed toes. Atrophy of the forearm and intrinsic hand muscles result in a claw hand. The proband (arrow) is a heterozygote for the p.G73D variant; WT, wild type. (**B**). Thoraco-lumbo-sacral MRI scan reveals hypertrophy in the intradural nerve roots of lumbar spine as well as cauda equina and brachial plexus (arrows). **Left panel**: T2-weighted sagittal and coronal sections, **right panel**: T1-weighted coronal and axial sections.

**Patient 1**: A 14-year-old female patient presented with walking difficulty in childhood. She noticed insidious muscle weakness in both the upper and lower extremities starting at the age of 10 years, which slowly accompanied symmetric muscle atrophy [19]. Physical examination at the age of 14 years revealed a steppage gait with worsening distal weakness and muscle atrophy in all extremities. Muscle atrophy was more pronounced in her upper and lower limbs (Figure 1A).

The NC study suggested demyelinating neuropathy. A bilateral pes cavus deformity was surgically corrected at the age of 11 years. She also developed a bilateral sensorineural hearing loss of 50 decibels, as reported for other CMT-DIE individuals [4]. Pathologic examination with a biopsied sural nerve did not reveal any remarkable proliferative changes. Persistent proteinuria manifested at the age of 11 years. At the age of 14 years, when she developed SRNS, the renal biopsy revealed FSGS histology. She progressed to ESRD at the age of 16 years (Table 1). At around the age of 30 years, she first noticed multiple subcutaneous nodules along the peripheral nerves of the cervical brachial plexus. Magnetic resonance imaging (MRI) scans with the thoracolumbar spine revealed hypertrophy in the intradural nerve roots of the lumbar spine, cauda equina, and brachial plexus with multiple nodules (Figure 1B).

**Figure 2:**
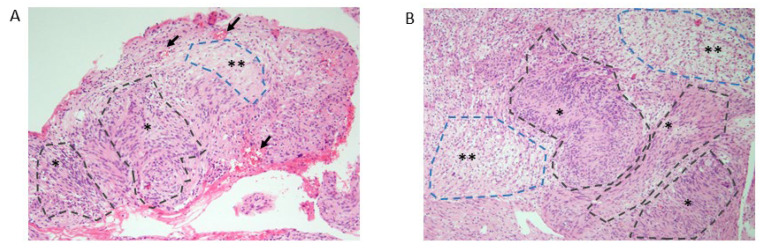
Histology of the cervical nerve tumors in CMT–FSGS patient 2 with INF2 p.V108D variant. Histology of surgically removed cervical mass at the age of 29 years revealed a biphasic pattern with mixed hypercellular (Antoni A, asterisk) and hypocellular (Antoni B, double asterisk) areas. Antoni A areas show a variably cellular lesion composed of spindle cells with focal nuclear palisading. Antoni B areas show a loose reticular or myxoid pattern, probably reflecting the degeneration form of the Antoni A area. Vascularization can be observed in the subcapsular areas (arrows). Cells composing the tumor do not show any mitotic activity or necrosis. (**A**) Lower magnification ×40, (**B**) higher magnification ×100. Hematoxylin and eosin stain.

**Figure 3:**
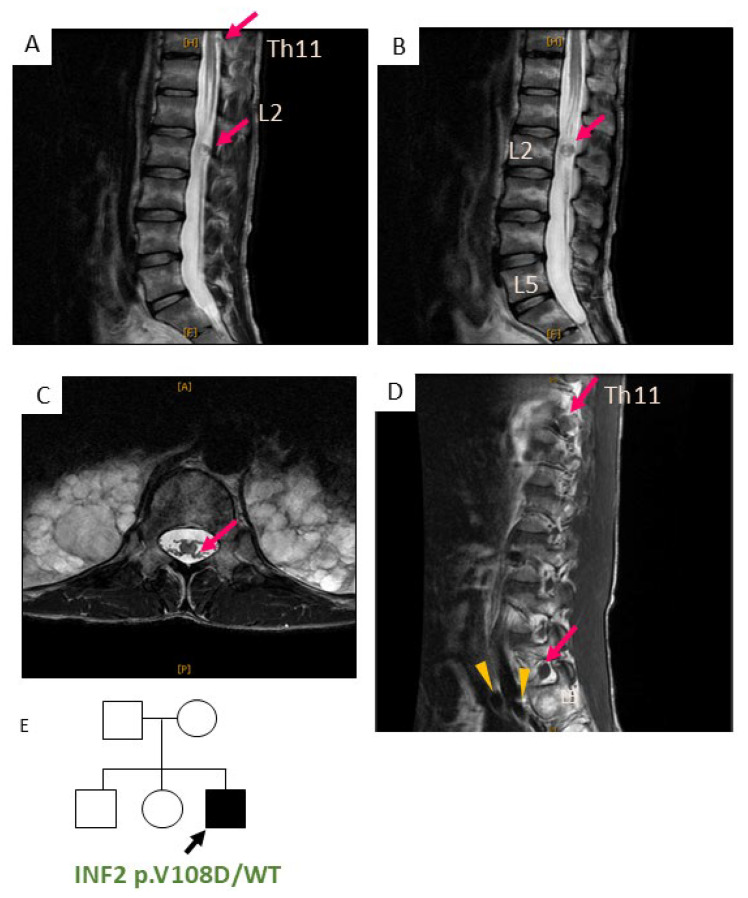
Spine MRI showing nerve hypertrophy of CMT–FSGS patient 2 with INF2 p.V108D variant. T2-weighted images of thoraco-lumbo-sacral spine MRI scan are shown for patient 2, who harbors INF2 p.V108D variant. (**A**,**B**) Sagittal scans revealed the thickened nerve roots at levels of Th11 and L2 (**A**) as well as an intradural, round mass at the L2 level inside the thecal sac (**B**). (**C**) Axial scan depicts intradural, root enlargement at the L2 level. (**D**) T1-weighted sagittal scan demonstrates the enlargement of the nerve roots (L3-S1 level) in both the intraforaminal (arrows) and extraforaminal regions (arrowheads). Hypertrophy was often observed for the nerve root at the exit from the thecal sac in the vicinity of the foramens. Some masses measured >1 cm in transverse diameter, where the normal range is from 2 to 3 mm [21,22]. (**A**–**C**) At the age of 32 years, (**D**) at age of 38 years. (**E**) Pedigree of patient 2. The proband (arrow) is a heterozygote for p.V108D. WT, wild type.

**Patient 2**: A 17-year-old male patient first noted a walking disability with the onset at the age of 10 years [20]. The NC study at the median nerve indicated a demyelinating subtype (Table 1). The biopsy of the sural nerve at the age of 19 years revealed degeneration in the large myelinated fibers, surrounded by onion bulbs. The patient first experienced proteinuria at the age of 12 years and developed SRNS at the age of 14 years. An open-kidney biopsy at age 17, when the eGFR was 24 mL/min/1.73 m^2^ with proteinuria of 6.8 g per day, revealed histology of a not-otherwise-specified (NOS) subtype of FSGS. Light microscopy showed global segmental sclerosis involving many glomeruli, predominantly in the juxtamedullary nephrons with tubular atrophy and interstitial fibrosis (Supplementary Figure S1). A cluster of tubular microcysts was seen in the cortex of the kidneys. Under higher magnification, podocyte hyperplasia and extracapillary cell proliferation mimicking a crescent were observed. The patient gradually progressed into ESRD at the age of 17 years (Table 1). Around that time, he noticed subcutaneous nodules in his neck and left lower extremity [20]. At the age of 29 years, he underwent surgical resection for cervical masses. The histologic examination of the surgical specimens revealed a feature of Schwannoma, as evident by palisading nuclei with hypercellular (Antoni A) and loosely arranged spindle cells in abundant myxomatous matrix areas (Antoni B) (Figure 2). Moreover, vascularization was observed in the subcapsular areas, and tumor cells showed no mitotic activity or necrosis. MRI scans of the spine revealed multiple round-shape masses and diffuse thickening of nerve roots at the spinal levels of cervical (C5/6), thoracic (Th11/12), limbo-sacral (L3 to S1), and cauda equina (Figure 3). Some hypertrophic roots were observed in intradural or foraminal regions, while others were found in the extradural (extraforaminal) space alongside the pelvic cavity wall and iliac muscles.

**Figure 4:**
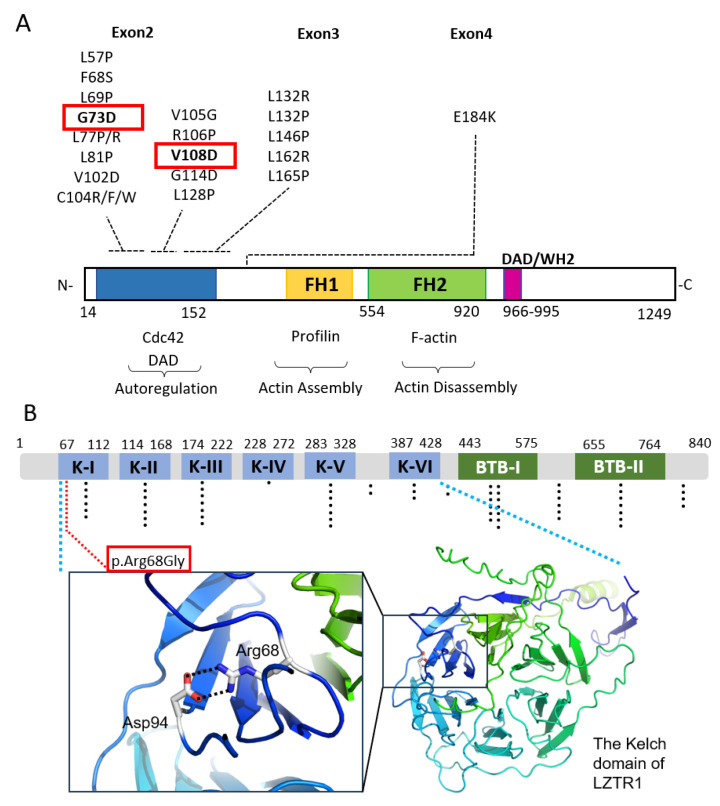
Locations of mutations of *INF2* and *LZTR1* in our CMT–FSGS cases. (**A**) The domain structure of *INF2* and locations of variants. The DID domain, formin homology domains (FH1, FH2), and the DAD domain are shown. The genetic variants previously reported in dual CMT–FSGS phenotype are shown above the domain structure. The variants of our present cases are boxed. The function of the distinctive domains and interacting partners are shown below the domain diagram. Amino acids are numbered according to NM_022489.4. Domains are defined by the NCBI Conserved Domains search base upon NP_071934.3. DAD: C-terminal diaphanous autoregulatory domain; DID: diaphanous inhibitory domain, FH1: formin homology 1; FH2: formin homology 2. (**B**) The domain structure of LZTR1 and locations Arg68Gly of variants. K-I~K-VI, Kelch motifs of the Kelch domain; BTB-I and BTB-II; BACK-I and BACK-II (partial BACK) domains. cDNA and amino acid positions according to NM_006767.4 and NP_006758.2. The positions of mutations previously identified in the schwannomatosis/glioblastoma are plotted with dots. There are no obvious cluster of mutations [23]. The location of p.Arg68Gly is highlighted with a red box. The Arg68 residue forms an ion pair with Asp94 in the β propeller of the first Kelch repeat domain, which forms the binding pockets for the substrates like RIT1. The structure of LZTR1 was predicted by AlphaFold3.

Sequencing of the *INF2* exon 2 revealed heterozygous, missense variants p.Gly73Asp (c.218G>A) and p.Val108Asp (c.323T>A) in patients 1 and 2, respectively (Figure 4A). Of note, these variants were absent in databases of Japanese healthy controls, namely the Human Genetic Variation Database (HGVD, *n* = 1208 individual) and the Integrative Japanese Multi Omics Reference Panel (MORP, jMorp 8.3K JPN). The amino acid positions Gly73 and Val108 are well conserved among vertebrate species and are classified as likely pathogenic (LP) based on the ACMG criteria (Supplementary Table S1).

Whole-exome sequencing with the genomic DNA from peripheral blood leukocytes showed that patient 1 with *INF2* p.Gly73Asp had no germline, schwannomatosis-related gene variants including *22q* loss of heterozygosity (LOH), *NF1*, *NF2*, *LZTR1*, *SMARCB1*, and *COQ6* (Supplementary Table S2, Supplementary Figure S2). In contrast, patient 2 with *INF2* p.Val108Asp had a concurrent germline variant of *LZTR1* p.Arg68Gly, while no schwannomatosis gene variants were found in patient 1. In silico analyses predicted that the *LZTR1* p.Arg68Gly variant has a damaging effect (Figure 4B, Supplementary Figure S3). This variant has not yet been reported in any other schwannomatosis patients or in ClinVar or other databases (OMIM, gnomAD, ClinGen, and Uniprot Variants) (Supplementary Table S1) and thus was scored as a variant of uncertain significance (VUS); there was neither LOH in the chromosome 22q lesion nor other mutations in the schwannomatosis-related genes [24,25]. The structural analysis predicted that the Arg68 to Gly substitution disrupts an ion pair with Asp94 in the first Kelch repeat domain, which likely compromises the size and shape of the binding pocket interacting with the substrate (RIT1) [18,26]. Patient 2 had other variants, Notch1 and 2 (Supplementary Table S1), previously reported in schwannomatosis patients but scored benign based on the ACMG criteria [17,27].

## 4. Discussion

We, for the first time, report a clinical appearance and pathogenic mechanism of nerve enlargement in two unrelated CMT-DIE patients with INF2 variants. Both individuals developed multifocal, hypertrophic changes in the nerves of the roots, trunks, and plexus with advance in the disease after the age of 30 years. Nerve enlargement has been reported for 25% of demyelinating CMT, the CMT1 subtype, which is the most prevalent subtype (~50% of all CMT) and is due to *PMP22* duplication. However, little is known about the *INF2*-related subtype (CMT-DIE) caused by INF2 variants (Supplementary Tables S3 and S4). Our two unrelated patients with the proximal *INF2* mutations took a similar clinical course. They manifested walking disability at nearly the same age of onset of renal disease (age 10 years) and multifocal nerve hypertrophy with advance of the disease (age 30 years). Therefore, the nerve enlargements of the two patients appeared to share the mechanism through which INF2 variants might alter the proliferation and remodeling activity of Schwann cells. Notably, the nerve hypertrophy was more pronounced in patient 2 than in patient 1, indicating that the germline schwannomatosis LZTR1 variant, despite the uncertain pathogenicity (VUS) at this moment, may contribute to hypertrophy changes. Our data suggest that the germline and/or somatic variants in schwannomatosis and/or neurofibromatosis genes may enhance the pathogenic effects of INF2 variants [25].

### 4.1. Mechanisms Through Which INF2 Variants Cause Glomerulopathy and Neuropathy

Our patients with dual CMT–FSGS, both of whom have the proximal INF-2 DID variants, also had severe renal disease with early-onset SRNS (at the age of 10 years) and rapidly progressed to ESRD (by the age of 20 years) (Supplementary Figure S4). This clinical manifestation contrasts that of patients with distal INF2-DID variants, who typically display milder renal phenotypes (a single FSGS disease). The observations suggest that dual CMT–FSGS variants compromise the organelle integrity in Schwann cells and podocytes more profoundly than FSGS single-disease variants [14]. Both our patients first experienced muscle weakness and an electrophysiological demyelinating feature in the first decade of life, as previously reported for INF2-related CMT [4,20,28,29]. Patient 2 showed an onion bulb formation in the biopsied sural nerves, while patient 1 had no remarkable proliferative changes. Such hypertrophic nerves may reflect Schwann cell proliferation in response to “dysmyelination” [30].

INF2 organizes the dynamics of actin and microtubule interactions in the cytoplasm [31]. Moreover, INF2 plays a key role in shaping glomerular slits and Schwann sheath and regulate their repair and remodeling. Prior human and rodent CMT model studies have shown that the repetitive injury of Schwann cells (demyelination) and repair (remyelination) might underlie the hypertrophic nerve changes in CMT (Supplementary Figure S5). Such aberrant proliferation might be inter-related with demyelination, augmenting the injury in Schwann cells. We did not specifically measure the cell proliferation rate in Schwann cells expressing the INF2 variants. However, previous studies revealed that demyelination and remyelination are closely associated with Schwann cell proliferation [32,33,34]. Indeed, the number of onion bulbs in sural nerve biopsy correlates with the occurrence of nerve enhancement and thickening in patients with lumbosacral MRI in CMT [35].

**Figure 5:**
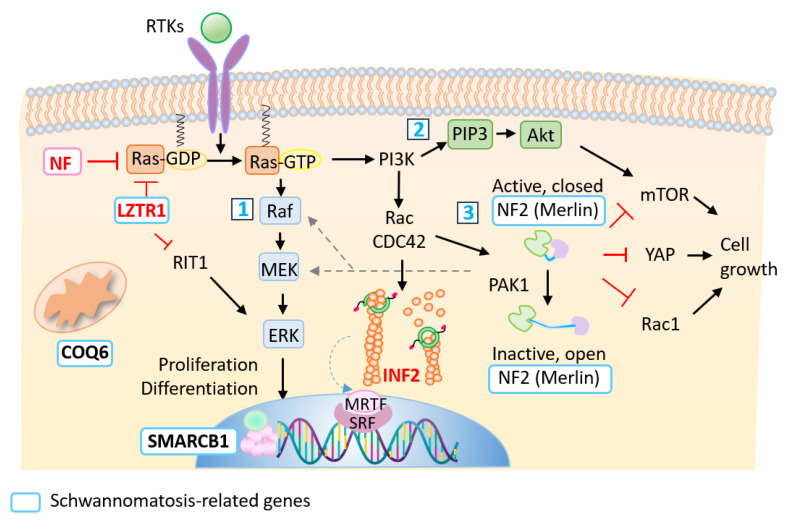
Regulatory pathways controlling cell proliferation and differentiation. The figure illustrates the signaling flow and crosstalk, together with positive (black arrows), dotted line or negative (red) control for the cascades. Classically, two tumor suppressors, NF1 and NF2, have been implicated in the peripheral nerve sheath tumors. NF1 (neurofibromin) converts active Ras (GTP-bound) to inactive Ras (GDP-bound). Activated GTP-Ras increases cell growth by evoking mitogen-activated protein kinase (MAPK) signaling (also known as the Ras–Raf–MEK–ERK pathway, box 1) as well as PI3K–AKT–mTOR signaling (box 2) [38,39,40,41]. These signal pathways mediate a wide variety of cellular functions, including cell proliferation, survival, and differentiation. NF2 (Merlin), box 3, is implicated as a negative regulator of Rac1, mTOR, and Hippo/YAP signaling, as well as the MAPK signaling pathway, the activation of all increases cell growth [42]. Recent genetic studies have shown that mutations in *LZTR1*, *SMARCB1,* and *COQ6* cause schwannomatosis. Loss of LZTR1 functions decrease the ubiquitination of Ras, thereby overactivating MAPK signaling. Dysregulation of the Ras–MARK signaling pathway presents clinically as a set of disorders of RASopathy (NF1, Noonan syndrome, etc.) [43]. A Ras GTPase, RIT1, has emerged as a driver of human diseases, i.e., Noonan syndrome and cancer. The mechanism is ascribed to the inability of RIT1 variants to interact with LZTR1, which hampers the protein degradation of RIT1 [44,45]. INF2 elongates and severs the linear F-actin filaments. INF2 also controls gene transcription by facilitating the nuclear transition of MRTF and activating the transcription of serum response factor (SRF) target genes, which encode structural and regulatory effectors of actin dynamics [46]. Abbreviations: PI3K, phosphatidylinositol 3-kinase; RTK, receptor tyrosine kinase; YAP, yes-associated protein; mTOR, mammalian target of rapamycin; PAK1, p21-activated kinase 1; LZTR1, leucine-zipper-like transcriptional regulator 1; RIT1, GTP-binding protein Rit1; SRF, serum response factor, MRTF, myocardin-related transcription factors.

In previous studies, INF2 R218Q knock-in mice lacked an apparent glomerular phenotype under physiologic conditions but could not reconstitute the slit diaphragm during the recovery phase of glomerular injuries [36]. Cells transfected with the INF2 variants alter a G-actin-mediated, serum-response factor (SRF) transcriptional machinery, which may hamper actin remodeling in the recovery process after tissue injury (Figure 5) [37]. We previously showed that INF2 variants also perturb the morphology and distribution of organelles by disorganizing the actin filaments and the microtubule network [4,5,6,14]. These observations suggest that INF2 variants impair the maintenance of the differentiation state and remodeling activity in Schwann cells and podocytes. Further studies will identify a potential therapeutic target implicated in Schwann cell plasticity, facilitating peripheral nerve repair after injury.

### 4.2. Clinical Features of Hypertrophied Nerves in CMT Caused by INF2 Variants

Both patients with DID-*INF2* mutations developed multiple hypertrophy along their peripheral nerves in the extremities (trunk, cervical brachial plexus), the spinal cord roots, and cauda equina, indicating a multifocal nature of Schwann cell proliferation. Nerve hypertrophy manifests in the advanced, later stage of the disease and becomes remarkable after age 30 years. In patient 2, nerve hypertrophy was more pronounced in intraforaminal spinal nerve roots at the cervical C5-6 and lumbosacral L3-S1 levels. In contrast, the thoracic spine was spared from the root enlargement, suggesting that Schwann cells may proliferate preferentially at the locations where the nerve roots more frequently experience mechanical stress. Most compression radiculopathy occurs at the cervical and Th12 to S1 roots after age 40 [21].

In our patients, hypertrophic nerves are depicted as low intensity masses on both T1- and T2-weighted imaging. The characteristics are consistent with those reported in the previous literature on CMT1A and CMT-DIE [12,35,47]. The MRI pattern may be heterogenous depending upon the disease chronicity, inflammatory responses, and environmental influence of the nerve lesions. The contrast CT or MRI images may help to characterize the vascularity of the nerve enlargement. Benign schwannomas are frequently enhanced on contrast imaging, as reported in CMT1A and schwannomas [11,12]. The nerve degeneration primarily affects the distal peripheral motor nerve as well as the nerve–muscular junction, preferentially involving the lower limb over the upper limb. We observed the typical physical manifestations of INF2-related CMT for our patients including atrophy of the forearm and intrinsic hand muscles (so-called “claw-hand”). Moreover, the atrophy of the leg muscles results in pes cavus and champagne-bottle deformity. The disability and muscle weakness due to neurodegeneration in the arm and foot comprise the core phenotypes of the CMT-DIE patients. The localized hypertrophic nerve mass may occasionally cause compression syndrome, affecting the spinal cord and cauda equina. This may manifest as various types of neuropathies, including radiculopathy, myelopathy, and cauda equina syndromes [21]. The observations suggest that the accumulating stress with age could exacerbate nerve enlargements. In our case, however, there was no significant difference in the daily life activity between patients 1 and 2: they could walk by themselves with crutches. Therefore, the severe hypertrophy of patient 2 did not simply correlate with the extent of mechanical stress overload.

Clinically, such focal hypertrophy and thickening of multiple nerves are observed under several conditions, including tumor syndrome, inflammatory disorders, infection, multiple sclerosis, chronic inflammatory demyelinating polyneuropathy (CIDP), and extracellular depositions (amyloidosis) [48,49]. Hypertrophied nerve roots have been reported for patients with CMT using pathological, ultrasonographic, and MRI imaging (Supplementary Tables S3 and S4) [50,51,52]. Ultrasonographic examination has become an essential tool for evaluation. The distribution pattern of hypertrophic lesions in CMT is generally diffuse and homogenous, while that in CIDP is regional and inhomogeneous [53]. Such morphological differences must be further evaluated in larger cohorts with sufficient observation periods under the unified diagnostic criteria.

Previous studies suggested that nerve enlargement is most often observed for a demyelinating subtype of CMT1, supporting the view that the primary demyelination could trigger the subsequent Schwann cell proliferation (Supplementary Figure S5). Nerve hypertrophy in patients with CMT may represent an initial CMT manifestation that precedes electrophysiological abnormalities [3,54]. Disorders primarily affecting Schwann cells lead to a loss of myelin, so-called segmental demyelination. The reactive Schwann cell hyperplasia surrounding the axons enlarges the affected nerves, which may become clinically recognizable as ”hypertrophic neuropathy”.

In adult CMT1A, multifocal hypertrophy is observed most commonly for the limb nerves as well as for the spinal cord, cauda equina roots, and cranial nerves on rare occasions [3,10] (Supplementary Tables S3 and S4). Previous studies on CMT1A showed that the severity of root hypertrophy is associated with the causative *PMP22* gene dosage as well as the frequency of onion bulb formation in biopsied nerves [10]. The excess of concentric proliferation onion bulb formation reflects segmental demyelination and parallels axon and Schwann cell degeneration [55]. So far, a correlation between onion bulb formation and nerve hypertrophy has been typically observed for CMT1A caused by *PMP22* duplication in 70% of cases and, to a lesser degree, in the HNPP subtype due to *MPZ* deletion [21,48,51,56]. Recent studies with human and mouse CMT models suggest that CMT1A might be “dysmyelination” disorder instead of demyelination, where myelination is delayed and hardly recovers to normal levels [57].

### 4.3. Roles of Schwannomatosis-Related Genes in Nerve Enlargement

The co-occurrence of CMT with schwannomatosis has so far been reported for five rare cases: three CMT1A, one HNPP, and one CMT4B1 (Table 2) [11,12,58,59]. However, the mechanisms remain unclear because of the lack of genetic analysis for the schwannomatosis genes. Previous studies have reported several rare cases in which CMT co-occurs with neurofibromatosis, accompanying the typical stigmata of café-au-lait spots (Supplementary Table S5). Four NF1 cases had concurrent CMT mutations, indicating the co-incidence of rare bigenic events. The remaining four NF1 cases had not undergone genetic testing for CMT. CMT with NF might be causally related, but it remains unclear whether or not environmental factors or other modifier genes may influence the nerve sheath tumor phenotype.

Nerve sheath tumors such as NF and Schwannoma are common, with the disease prevalence being 1:3500 for NF1 and 1:50,000 for NF2. In this context, it might be possible that these peripheral nerve sheath tumors incidentally co-occur with demyelinating CMT [13]. Both patients 1 and 2 had no family history or the skin, ocular, or vestibular manifestations, the stigmata of the *NF1*, and *NF2*-related disorders. The nerve enlargements in multiform neurofibromatosis usually show a more nodular appearance [57]. Moreover, we excluded mutations of the *NF1* and *NF2* gene, or 22q LOH, via the whole-exome sequencing of the genomic DNA of the peripheral blood from patients 1 and 2. The absence of the bilateral vestibular Schwannoma in both patients argued against the diagnosis of *NF2* (Supplementary Table S2). Specifically, the biphasic pattern characterized by the hypercellular (Antoni A) and hypocellular (Antoni B) regions represent typical Schwann cell proliferation and degenerative changes, respectively. The typical biphasic pattern is characteristic of Schwannoma and excludes the possibility of other nerve sheath tumors like neurofibroma. According to the recent guidelines, patient 2 met the clinical/genetic diagnostic criteria for schwannomatosis: biopsy-proven Schwannoma with the germline LZTR1 variant [60,61].

### 4.4. Limitations

Our study provided histological and radiological evidence of nerve enlargement in INF2-related CMT. However, detailed characterization of hypertrophy, such as ultrastructural and/or higher magnification analyses, was not conducted. Additionally, we did not fully evaluate the serial electrophysiological profile or the histological and radiographic changes in the peripheral nerves throughout the course of the disease. We did not analyze the somatic mutations in the peripheral nerve masses, which would be necessary to test the multiple-hit hypothesis [25]. This study is based on two cases of CMT and FSGS, which reflects the condition’s rarity. While the findings provide valuable insights, the limited sample size restricts the generalizability of the results. Future studies with a larger cohort and incorporating advanced histological and radiological evidence would help to verify our multigene model underlying Schwann cell proliferation.

While this study focused on the genetic and clinical characteristics of neuropathy, environmental factors, such as food, living conditions, and occupational exposure, may influence the occurrence and progression of CMT–FSGS diseases.

## 5. Conclusions

Our two patients with INF2-related CMT showed peripheral nerve hypertrophy in adulthood. The observations support that the enlargement of the nerve roots might be due to Schwann cell proliferation in response to demyelination and “dysmyelination” over the year. Defective actin remodeling by INF2 variants may be linked to the aberrant proliferative response of Schwann cells. Schwannoma could co-occur in patients with INF2-related CMT, particularly in whom the germline schwannomatosis-predisposing variants such as *LZTR1* (Supplementary Figures S2 and S3) simultaneously exist. The relevance of the germline *LZTR1* variants in patient 2 must be interpreted by taking into account the allelic status of somatic variants in tumor tissues. A larger cohort is necessary to clarify the roles of the predisposing genes (such as somatic LZTR1) in Schwann cell proliferation. The enlargement of the peripheral nerves might have been underevaluated and may happen more often than we previously thought. Further studies with hypertrophic features will help discover the molecular mechanisms underlying schwannoma tumorigenesis and neuropathies and improve therapeutic strategies in INF2 disorders.

## Figures and Tables

**Table 1:** Clinical characteristics of CMT–FSGS patients with INF2 variants.

Case	Sex	Renal Phenotype				Neurological Phenotype				Other Features
		Onset Age (Year)			Renal Histology	Onset Age Walking Disability (Year)	Electrophysiology		*PMP22* Copy Number ^(a)^	
		Proteinuria	NS	ESRD			Subtype	NCV m/s		
1	F	11	14	16	FSGS	10	Demyelinating	No potential	Wild type	Hearing loss
2	M	12	14	17	FSGS	10	Demyelinating	No potential	Wild type	Onion bulb formation ^(b)^

M: male; F: female; NS: nephrotic syndrome; ESRD: end-stage renal disease; FSGS: focal segmental glomerulosclerosis; NCV: nerve conduction velocity. Demyelinating and axonal subtypes were defined by traditional electrophysiological criteria, NCV < 35 m/s and NCV > 45 m/s, respectively, whereas the normal range is 40–45 m/s [3]. ^(a)^ Duplication of peripheral myelin protein (*PMP22*) on X chromosome 17p12, a genetic defect of CMT1A, representing the most prevalent form (~50%) of all CMTs, was screened by fluorescence in situ hybridization (FISH). ^(b)^ Pathological study with biopsied sural nerve at age 19 years revealed a loss of large-caliber, myelinating fibers and concentric hyperplasia of Schwann cells (onion bulb formation).

**Table 2:** Summary of previous reported cases of peripheral neuropathy with concurrent Schwannoma.

	Reference	Neuropathy			Schwannomas		CMT Mutation	Other Features
		Subtype	Age Onset (Year) Sex	Initial Manifestation	Age Onset (Year)	Affected Location		
1	Kwon et al., 2009 [12]	CMT-1A	9, M	Walking difficulty	14	Spinal cord (T12-L1) Median nerve	*PMP22* duplication	No mutations in *NF1* or *NF2*
			20, F	Weakness in both lower extremities	25	Median Nerve	*PMP22* duplication	Mother of the proband ^(a)^
2	Heckman et al., 2007 [11]	HNPP	42, F	Pain of the left medial ankle radiating into the medial plantar foot	42	Median and medial plantar nerves	*PMP22* deletion	Clinically, absence of NF1 or NF2 manifestation.
3	Roohi et al., 1982 [58]	CMT	28, M	Leg weakness, NCV borderline	38	Cauda Equina T12-L1	*N.D* ^(b)^	painless cauda equina tumours, histologically schwannoma.
4	Scott et al., 2016 [59]	CMT4B1	9, M	Bilateral, symmetric limbs weakness in distal limbs	27	Cervical cord root (C2 and C3) Optic neuritis	*MTMR2*, c.1768C>T (p.Gln590Ter) ^(c)^	Only proband is affected with both CMT and schwannomas. No Schwannomas nor optic neuritis in other affected members

The previous reported cases, in whom histologically proven Schwannoma co-occurred with CMT, are summarized. ^(a)^ The segregation of genotype and phenotype suggests parent-to-child trans-mission. ^(b)^ Genetic testing was not performed. ^(c)^ The C>T substitution at nucleotide position 1768 (c.1768C>T) incorporates a premature stop codon at amino acid position 590. Abbreviations: NF, neurofibromatosis; CMT, Charcot–Marie–Tooth; HNPP, hereditary neuropathy with liability to pressure palsies; PMP, peripheral myelin protein; MTMR, myotubularin-related protein; NF1, neurofibromatosis type 1; NF2, neurofibromatosis type 2.

## Data Availability

The datasets used and/or analyzed during this current study are available from the corresponding author upon reasonable request.

## Supplementary Materials

The following supporting information can be downloaded at: https://www.mdpi.com/article/10.3390/biomedicines13010127/s1. Supplementary Figure S1. Renal Histology of the patient 2 harboring INF2 p.V108D variant. Supplementary Figure S2. Diagnostic Workflow for Schwannomatosis. Supplementary Figure S3. Sequence and structural analysis of LZTR1 variant. Supplementary Figure S4. Clinical course of our CMT-FSGS patients with the DID–INF2 variants. Supplementary Figure S5. Hypothetical pathogenesis model of hypertrophic nerve changes in demyelinating neuropathy. Supplementary Table S1. Germ-line variants identified by exome analysis in CMT-DIE patients 1 and 2. Supplementary Table S2. Revised diagnostic criteria for schwannomatosis with pathogenic SMARCB1 or LZTR1 variants. Supplementary Table S3. Case reports showing the nerve hypertrophy in CMT. Supplementary Table S4. Comparison of peripheral nerve enlargement among CMT and other demyelinating disorders. Supplementary Table S5. Case reports of peripheral neuropathy with neurofibromatosis. Supplementary References [62,63,64,65,66,67,68,69,70,71,72,73,74,75,76,77,78,79,80,81,82,83,84,85,86] were cited in the Supplementary Materials: [62,63,64] for Supplementary Figure S5, [65,66,67,68,69,70] for Supplementary Table S3, [71,72,73,74,75,76,77,78,79,80] for Supplementary Table S4, and [81,82,83,84,85,86] for Supplementary Table S5.
