# Supplementary material for: Nerve Enlargement in Patients with INF2 Variants Causing Peripheral Neuropathy and Focal Segmental Glomerulosclerosis
Source: Biomedicines. 2025 Jan 8;13(1):127. doi: 10.3390/biomedicines13010127 (PMC11763285; doi:10.3390/biomedicines13010127)
Supplement: Supplementary file 1 [file biomedicines-13-00127-s001.zip › R1 by HT supplementary Figures.pdf]

Nerve Enlargement in Patients with INF2 Variants Causing Peripheral Neuropathy and Focal Segmental Glomerulosclerosis

Quynh TH Tran, Linh NT Tran et al

Table of contents for supplementary Information

Supplementary item	Contents	Page
Supplementary Fig.S1	Renal Histology of the patient 2 harboring INF2 p.V108D variant	2
Supplementary Fig.S2	Diagnostic Workflow for Schwannomatosis	3
Supplementary Fig.S3	Sequence and structural analysis of LZTR1 variant	4
Supplementary Fig.S4	Clinical course of our CMT-FSGS patients with the DID–INF2 variants	5
Supplementary Fig.S5	Hypothetical pathogenesis model of hypertrophic nerve changes in demyelinating neuropathy	6
Supplementary Tab.S1	Germ-line variants identified by exome analysis in CMT-DIE patients 1 and 2	7
Supplementary Tab.S2	Revised diagnostic criteria for schwannomatosis with pathogenic SMARCB1 or LZTR1 variants	8
Supplementary Tab.S3	Case reports showing the nerve hypertrophy in CMT	9
Supplementary Tab.S4	Comparison of peripheral nerve enlargement among CMT and other demyelinating disorders	10
Supplementary Tab.S5	Case reports of peripheral neuropathy with neurofibromatosis	11

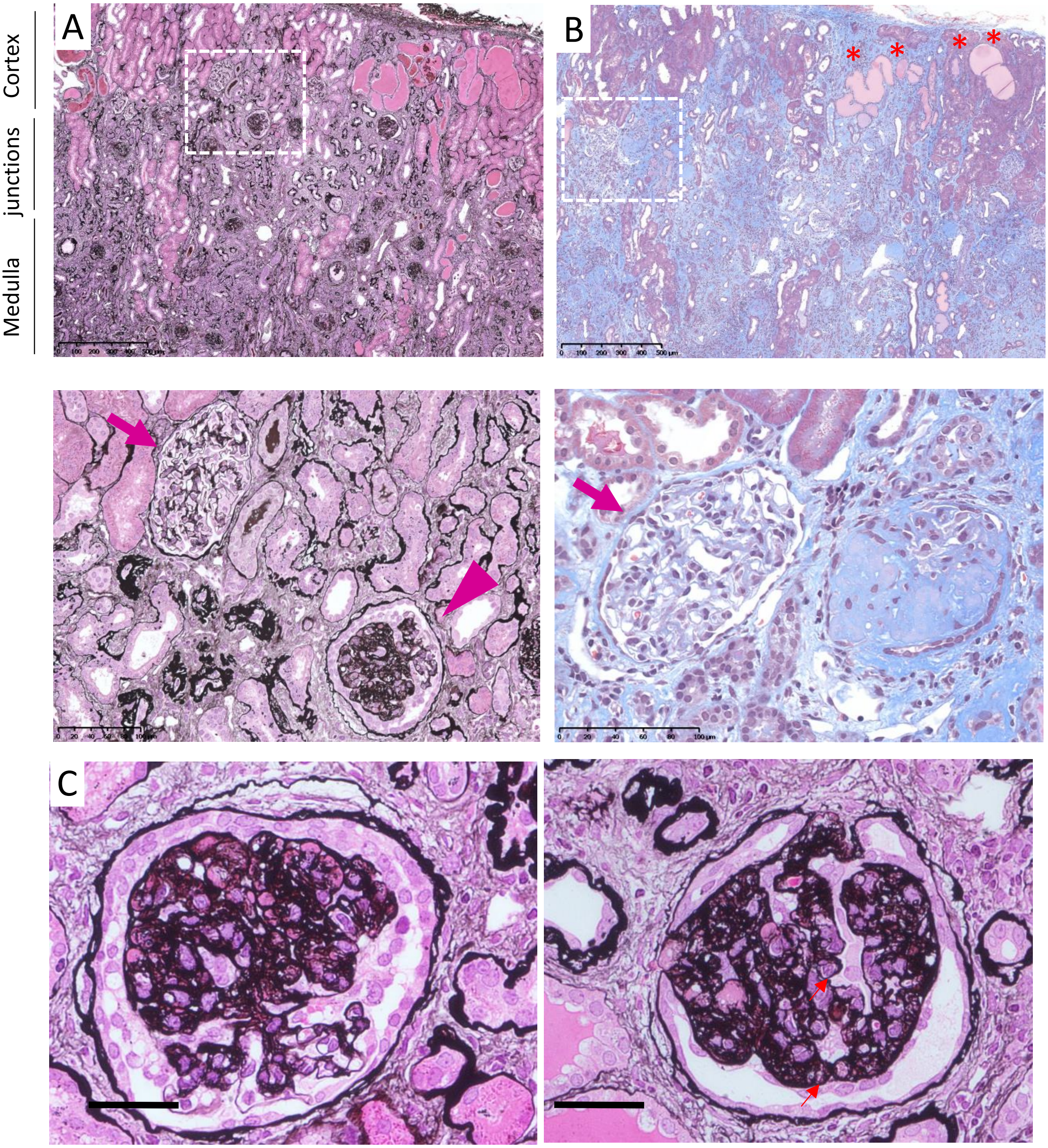

Supplementary Figure S1. Renal Histology of the patient 2 harboring INF2 p.V108D variant. Renal histology is shown for patient with INF2 p.V108D variant who underwent an open biopsy at age 17 when eGFR was 24.1ml/min with nephrotic range of proteinuria of 6.8 g per day. **A, B. Low magnification** Global glomerulosclerosis predominantly in juxtamedullary nephrons, zonal tubular atrophy, and interstitial fibrosis. Focal microcystic dilation of tubules is found in the cortex (asterisks, 5×). Moderate magnification is shown in the middle panel. Minimal-changes but hypertrophied glomeruli (arrows) were observed adjacent to the global sclerotic glomeruli (arrowheads) in the corticomedullary junctions (30×). Scale bars 500 μm in A and 100 μm in B. **C**. **Higher magnification.** Glomeruli show global sclerosis with adhesions to the Bowman's capsule. There are columnar cuboidal Bowman's capsule epithelia. Capillary lumen obliteration with residual podocyte hyperplasia (arrows) and hyaline material deposition. The tubular interstitium surrounding the glomeruli show marked fibrotic changes with tubular atrophy (50×). Scale bars: 50 μm. Periodic acid methenamine silver (A, C) and Masson Trichrome (B) staining are shown. eGFR: estimated glomerular filtration rate.

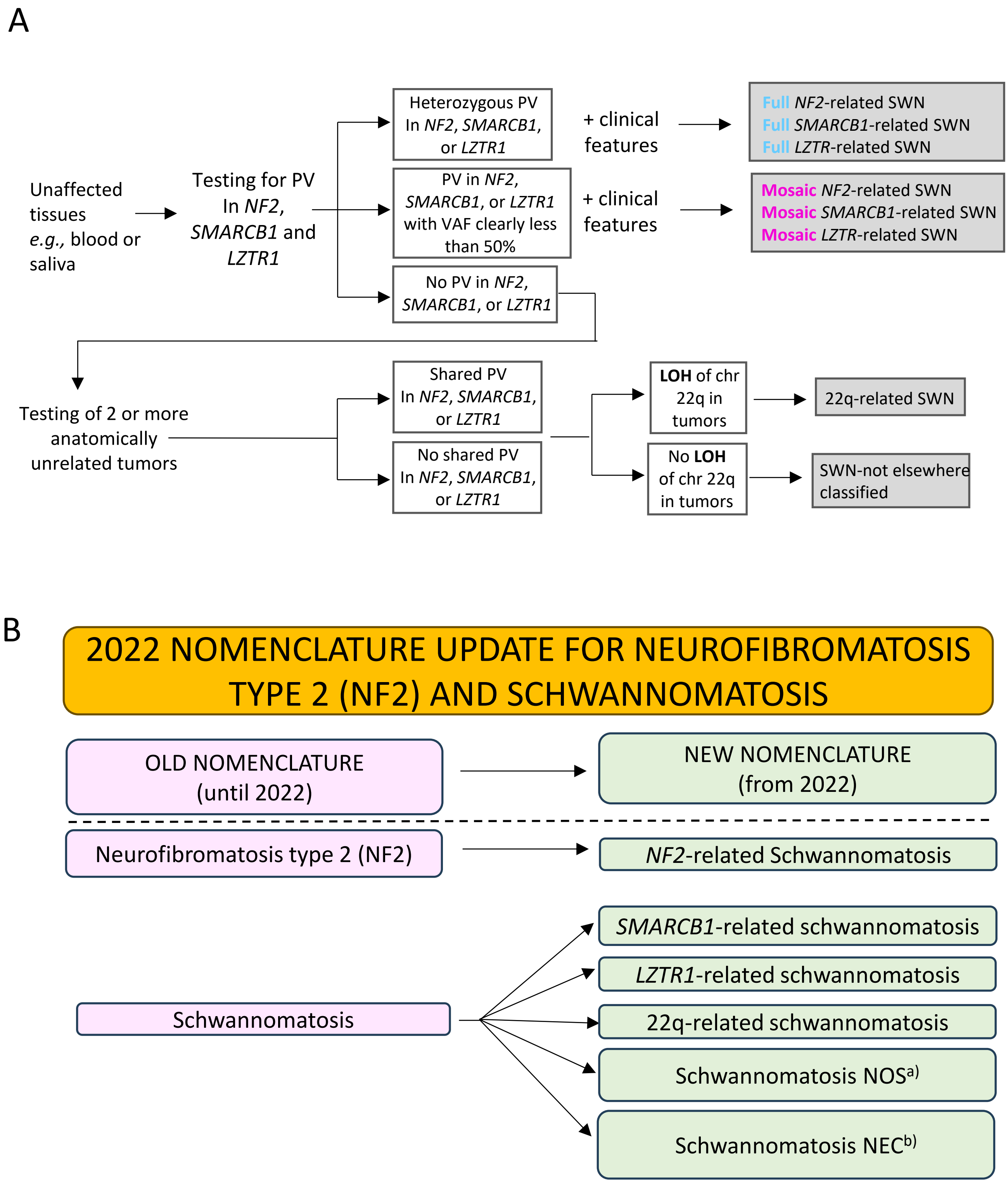

Supplementary Figure S2. Diagnostic Workflow for Schwannomatosis.
A. Flowchart of genetic testing strategy for neurofibromatosis NF2 and schwanomatosis (SWN). The schema is proposed by Plotkin SR, et al (Genetics in Medicine, 2022). LOH, loss of heterozygosity; NF2, neurofibromatosis type 2; PV, pathogenic variant; SWN, schwannomatosis; VAF, variant allele fraction.
B. New nomenclature of European Reference Network, Genetic Tumour Risk Syndrome (ERN GENTURIS). In the new nomenclature, “Neurofibromatosis type 2” is assigned as “NF2-related schwannomatosis”. “Schwannomatosis” is further categorized into five subtypes including “*SMARCB1*-related schwannomatosis”, “*LZTR1*-related schwannomatosis”, “22q-related schwannomatosis”, or “schwannomatosis not otherwise specified (NOS)”.

[a)] NOS (not otherwise specified) for patients who have not had the genetic testing.

[b)] NEC (not elsewhere classified) for patients in whom genetic testing of blood/saliva and tumors failed to detect a pathogenic variant.

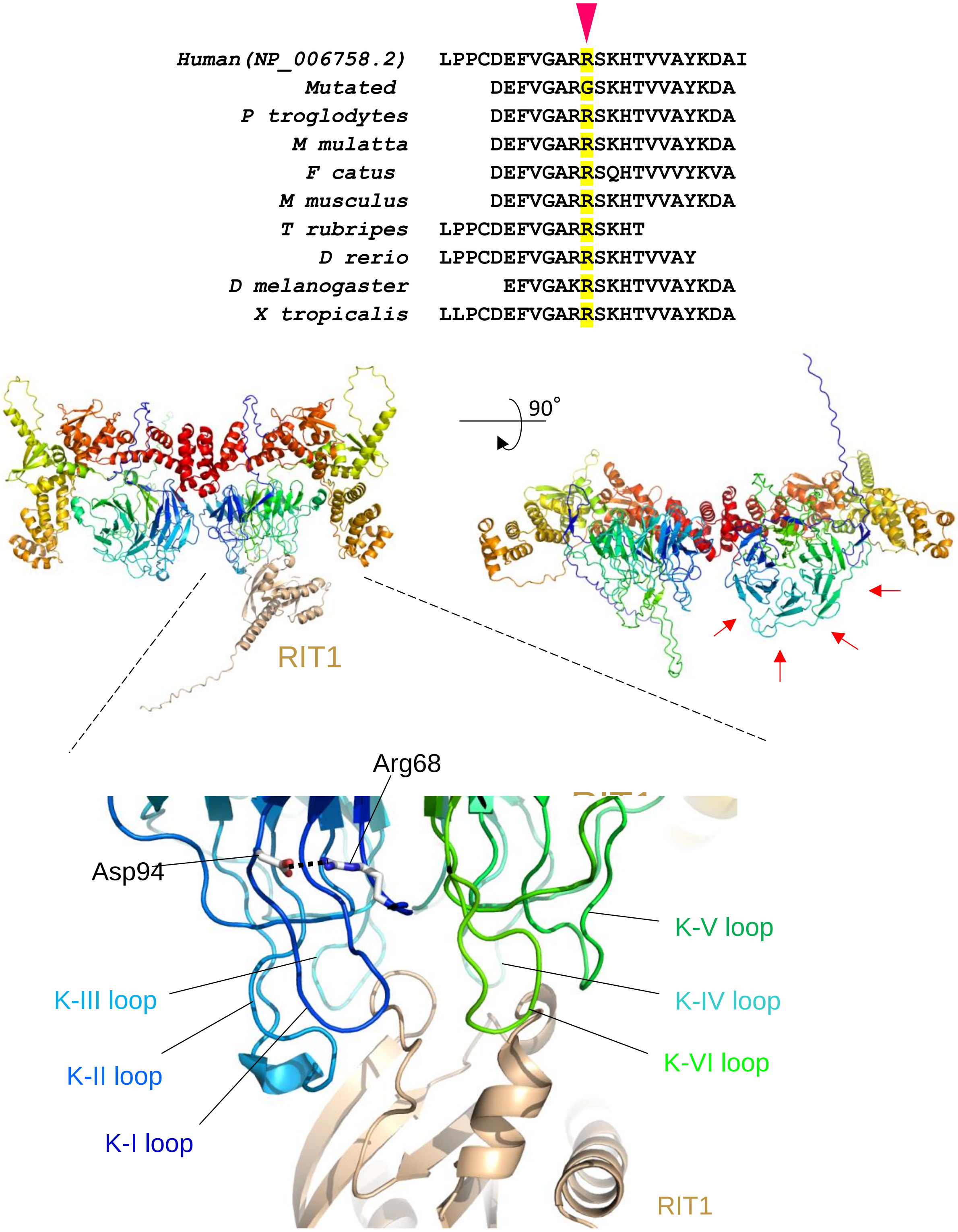

Supplementary Figure S3. Sequence and structural analysis of LZTR1 variants

A, Multiple alignment of LZTR1 orthologs. The Arg 68 residue is conserved across various species.

B, LZTR1 homodimer – RIT1 complex. Overview of the 3D structure predicted by AlphaFold3, is shown from the side and top (vertically rotated by 90°), showing an enface view of β propeller (arrows). Clustering of loop segments in each 6 Kelch repeat (K-I to K-VI) comprises a binding pocket with the substrates such as RIT1 (isoform 3, bottom).

C, Closed-up view of LZTR1–RIT1 interface. The structure is predicted by AlphaFold3. Arg68 to Gly substitution disrupts an ion-pair with Asp 94 in K-I loops, which form a binding site to the RIT1. LZTR: Leucine-zipper transcription regulator 1; RIT1: GTP-binding protein RIT1

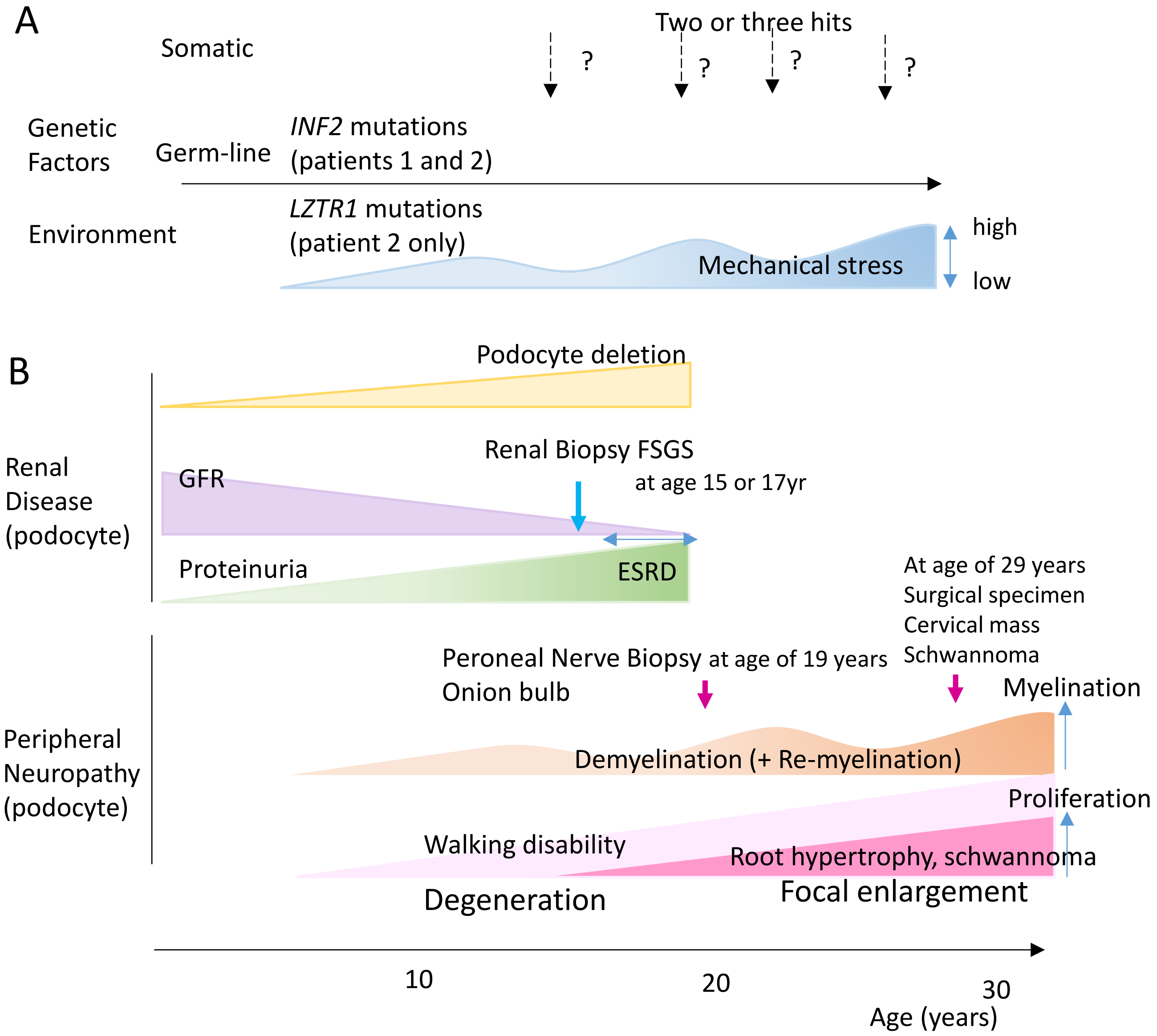

Supplementary Figures S4. Clinical course of CMT-FSGS patients with the DID–INF2 variants

A. Pathogenic factors of INF2 disorders. Both patients 1 and 2 have pathogenic DID *INF2* variants, p.G73D and p.V108D, respectively. Additionally, patient 2 harbours a *LZTR1* R68G VUS variants, while patient 1 has no schwannomatosis-related gene variants (*LZTR1*, *SMARCB1*, *22q* LOH). Somatic mutations (*LZTR1*, *SMARCB1*, *NF2, 22q* LOH, dot arrows) in Schwannomatosis-related genes, although it had not yet been studied for these patients, may contribute to the Schwann cell proliferation in an age-dependent manner. Non-genetic modifier (mechanical compression, inflammation, etc) may modify the clinical phenotypes.

B. Clinical course of patients with INF2 disorders. Patients with p.G73D, p.V108D show a dual disease phenotypes of glomerulopathy and peripheral neuropathy. Glomerulopathy is characterized by SRNS, which presented around age 10 years and progressed into ESRD at age 17-19 years. Renal histology showed FSGS in both patients. Peripheral neuropathy developed at nearly the same time with walking disability and muscle weakness. The electrophysiological NC studies indicate a demyelinating subtype. Peroneal nerve biopsy at age of 19 years reveal an onion-bulb formation in patient 2. Evolution of multiple nerve hypertrophy after age of 30 years correlates clinically with increasing neurological disability. The nerve enlargement likely reflects reactive Schwann cell proliferation in response to the de- and remyelination process.

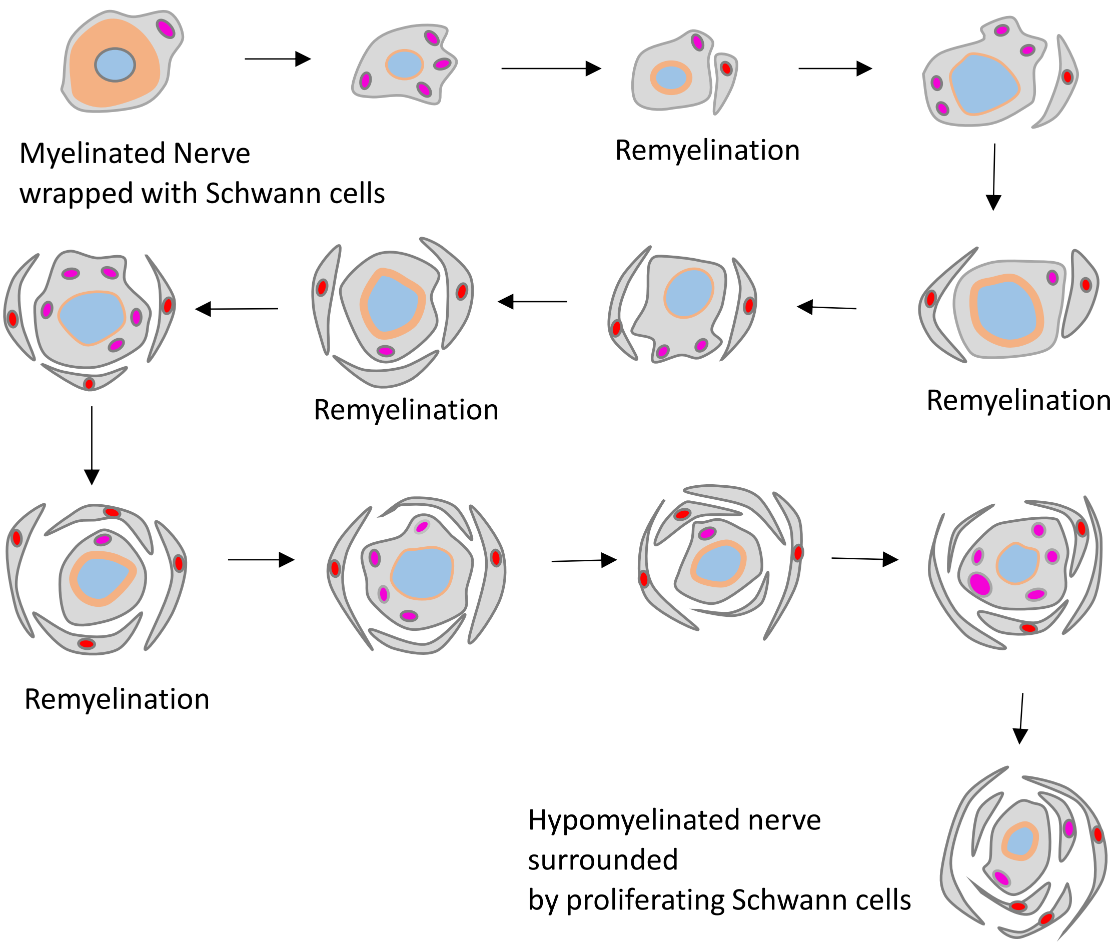

Supplementary Figure S5. Hypothetical pathogenesis model for hypertrophic nerve changes in demyelinating neuropathy.

Schwann cells seldom divide in normal adult nerves. Loss of the myelin sheath in disease leads to active proliferation of Schwann cells.

Pathognomonic findings of CMT1 (demyelinating type) rely on repeated cycles of demyelination and remyelination, which produces a concentric, multiple layer of Schwann cells around axons, the so-called “onion-bulb formation”. The axon is often present in the center of the onion bulb, and the myelin sheath is usually thin or absent [62, 63].

Clinically, the supernumerary layers of Schwann cell hyperplasia surrounding individual axons make individual peripheral nerves enlarged, which may be individually palpable, so called ”hypertrophic neuropathy” [35]. In the longitudinal plane, individual segments of the axon may display appearance of segmental demyelination. Studies with pediatric CMT1A disclosed that the onion-bulb formation increases markedly in number and size during later childhood [64].
