# Supplementary material for: Nerve Enlargement in Patients with INF2 Variants Causing Peripheral Neuropathy and Focal Segmental Glomerulosclerosis
Source: Biomedicines. 2025 Jan 8;13(1):127. doi: 10.3390/biomedicines13010127 (PMC11763285; doi:10.3390/biomedicines13010127)
Supplement: Supplementary file 1 [file biomedicines-13-00127-s001.zip › R1 by HT Supplementary Tables S1-S2-S5.pdf]

Supplementary Table S1. Germ-line variants identified by exome analysis in CMT-DIE patients 1 and 2.

Family	*Gene*	Amino Acid Change	Nucleotide change		Inheritance	gnomAD v4.1.0	Clin-Var	ACMG Criteria			References
			cDNA position	zygosity	(segregation)	MAF		Supporting evidence for pathogenicity	Codes	Class	
Case1	*INF2*	p.Gly73Asp	c.218 G>A	Hetero	*de novo*	0 *	NA	2 Strong,4 Moderate and 3 Supporting	PS 1,3, PM 1,2,5,6, PP2,3,4	P	Hara M, 1984, Barua M, 2013
Case2	*INF2*	p.Val108Asp	c.323 T>A	Hetero	*de novo*	0 *	LP	2 Strong, 2 Moderate and 2 Supporting	PS1, 3, PM1, 2, 5, PP2, 4	P	Toyota K, 2013
	INF2	p.Val854Met	c.2560G>A	Hetero	*de novo*	0.000020	LB	3 Supporting	PM2, BP1, BP4, BP6	LB	NA
	LZTR1	p.Arg68Gly	c.202C>G	Hetero	*de novo*	0 *	NA	1 Strong and 2 Supporting	PM2, PP3, BP1	VUS**	This study
	NOTCH1	p.Glu2254Lys	c.6760G>A	Hetero	*de novo*	0.000007	VUS	3 Supporting	PM2, BP1, BP4	LB	Nykamp K, 2017
	NOTCH2	p.Gly31Asp	c.92G>A	Hetero	*de novo*	0	NA	1 Strong and 2 Supporting	PM2, BP1, BP4	LB	NA

Exome sequencing was done by using the genomic DNA from the peripheral blood leukocyte.
The nucleotide positions of variants are according to the GenBank RefSeq: *INF2* NM_022489.4, *LZTR1* NM_006767.4, *NOTCH1* NM_017617.5, *NOTCH2* NM_024408.4.
Population allele frequency is referred to the public database: gnomAD: the genome Aggregation Database MAF, minor allele frequency.
Asterisks: These variants are absent from the database of Japanese healthy controls: HGVD, human genetic variation database; ToMMo, Japanese Multi-Omics Reference Panel (iMorp, 8.3K JPN) of the Tohoku University Tohoku Medical Megabank Organization (ToMMo, Nucleic Acids Research, 2023, doi: 10.1093/nar/gkad978).
Double asterisk: *In silico* analysis predict SIFT score =0 damaging; Polyphen-2 score =0.997, deleterious; Mutation Taster, Disease causing
Variants are classified according to the recommendations by the American College of Medical Genetics and Genomics (Richards et al., 2015; Nykamp et al., 2017): P, pathogenic; LP, likely pathogenic; VUS, Uncertain significance; LB, likely benign; B, benign; NA, not appreciable.

Supplementary Table S2. Revised diagnostic criteria for schwannomatosis with pathogenic *SMARCB1* or *LZTR1* variants

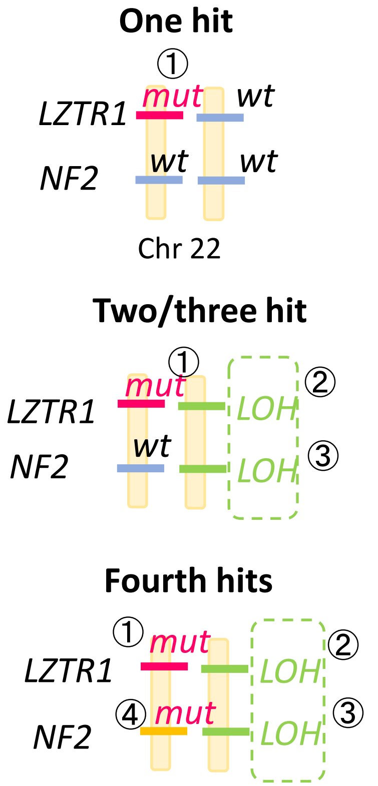

Diagnostic criteria for *SMARCB1*- and *LZTR1*-related schwannomatosis

A diagnosis of *SMARCB1*- or *LZTR1*-related schwannomatosis can be made when an individual meets one of the following criteria:

- At least one pathologically confirmed schwannoma or hybrid nerve sheath tumor and a *SMARCB1* (or *LZTR1*) pathogenic variant in an unaffected tissue such as blood[a)]
- A shared *SMARCB1* or *LZTR1* pathogenic variant in two schwannomas or hybrid nerve sheath tumors.

Pattern of genetic changes in unaffected and tumor tissue in *SMARCB1*- and *LZTR1*-related schwannomatosis[b)]

Gene locus	Unaffected tissue[c)]	Tumor1	Tumor2	Comments
SMARCB1/LZTR1				
Allele 1	PV1[d)]	PV1	PV1	Shared *SMARCB1* or *LZTR1* pathogenic variant
Allele 2	WT	LOH	LOH	Tumor-specific partial loss of 22q in *trans* position, LOH typically entails deletion of 22q region, encompassing *LZTR1/SMARCB1/NF2*
NF2				
Allele 1	WT	PV2	PV3	Tumor-specific pathogenic *NF2* variant in *cis* to pathogenic *SMARCB1* variants
Allele 2	WT	LOH	LOH	Tumor-specific partial loss of 22q in *trans* position, LOH typically entails deletion of 22q region, encompassing *LZTR1/SMARCB1/NF2*

LOH, loss of heterozygosity; PV, pathogenic variant; WT, wildtype. **a)** If a likely pathogenic variant is identified, tumor analysis may aid upward classification to pathogenic variant. **b)** A second somatic, contiguous chromosome 22q deletion including *LZTR1, SMARCB1,* and *NF2* occurs *in trans* with the first hit of *the* germ-line *SMARCB1* or *LZTR1* variants. **c)** Tissues unaffected by tumors such as blood or skin. **d)** If the variant allele fraction is clearly <50%, the diagnosis is mosaic *SMARCB1*- and *LZTR1*-related schwannomatosis.

The fact that somatic *NF2* mutations often coexist with germline SMARCB1 or LZTR1 mutations in schwannomatosis indicate that the classical 2-hit model of tumorigenesis is insufficient to account for the development of schwannomatosis. According to the 4-hit/3-step model (Kehrer-Sawatzki H 2018), patients harbour heterozygous, germline *LZTR1* (or *SMARCB1*) mutations (first hit). The counterpart allele of 22q, being *in trans* with the first hit, gets partially deleted (LOH), where the contiguous 22q deletion typically entails *LZTR1* (or *SMARCB1*) and *NF2(s*econd/third hit*)*. The remaining *NF2* allele further gains a somatically acquired tumour-specific mutation, *in cis* with the first *LZTR1/SMARCB1* hit (fourth hit). These observations indicate that tissue-specific *NF2* inactivation is crucial for the pathogenesis of *NF2*-related as well as *LZTR1* (or *SMARCB1*) schwannomatosis.

References: Plotkin SR et al, Genetics in Medicine (2022) 24, 1967–1977; Kehrer-Sawatzki H, Hum Genet (2018) 137, 543–552.

Supplementary Table S5. Case reports of peripheral neuropathy with neurofibromatosis.

Ref	Neuropathy			NF1 phenotype	CMT mutation	Others
	Subtype	Age of onset (year), gender	Initial symptoms			
Koc et al., 2009[81]	CMT1A	19, M	Progressive gait disturbance and leg weakness	Diagnosed with NF type 1 at the age of 9 years	*PMP22* duplication	
Lancaster et al., 2010[13]	CMT-1B	50, M	Progressive foot numbness	Cafe-au-lait macules and multiple subcutaneous neurofibromas in childhood. CMT score 12, NCV demyelinating 17-27 m/sec	*MPZ* c.449-1G>A (splice site)	Clinically diagnosed as NF1, but no genetic testing for *NF1*
Lupski et al., 1993 [82]	CMT-1A	18, M	Distal muscle atrophy and weakness, pes cavus, and absent deep tendon reflexes	Diagnosed with NF type 1 at age of 18 years with cafe-au-lait spots and cutaneous neurofibromas	17p12 duplications	CMT1A duplication inherited from their affected parents with CMT
		14, F	Weakness of all limbs, absent deep tendon reflexes in the legs, and bilateral pes cavus	Hyperpigmented on trunk, neck and arms, large mass in the cervical, spinal, left flank with pathologically indicated benign neurofibroma		
Onu et al., 2013 [83]	CMT 2	64, M	Weakness and numbness of the arms and legs, radicular pain in the lower cervical region bilateral C2 nerve root tumors	Café-au-lait macules, axillary freckles, and subcutaneous neurofibromas, diagnosed in aldolescence	N.D.	Diagnosed with CMT around age 50 on a clinical basis
Ma'luf et al., 2005 [84]	CMT	35, M	Bilateral exophthalmos, mild limitation of supraduction in both eyes. Orbital CT showed bilateral large masses filling the superior orbits, displacing the globes inferiorly	Neurofibroma arising from a peripheral nerve, wavy Schwann cells within the neurofibroma in biopsied orbital mass	N.D.	Co-occurrence of bilateral orbital tumors. No café-au-lait spots
Bosch et al., 1981 [85]	CMT Case 1	8,M	Walking difficulties, deformity of both feet	Café au lait spots at age 8 and subcutaneous neurofibroma on the left thigh at age 19.	N.D.	Onion bulb formation. NCV reduced borderline
	Case 2		Ankle instability	Neurofibromas developed early adult years. At age 57 years, neurofibromas and café au lait spots over the trunk	N.D.	Onion bulb formation, cluster of small myelinated fibers
	Case 3	29, F	Mild symmetric atrophy and weakness of tibialis anterior and peroneal muscles	Neurofibromas in adolescence, multiple café au lait over face, trunk and extremities	N.D.	
	Case 4	21, M	Progressive weakness and numbness of legs	Café au lait spots, axillary freckles, subcutaneous nodules over the trunk and limbs	N.D.	
Roos et al., 1989[86]	CMT + NF1	30, M	Lower extremeties muscle weakness, atrophy, sensory loss	hypertrophic lumbosacral nerve roots	N.D.	

CMT and NF1 each occur with an incidence of 1 in 2500, so both disease could co-exist as rare by chance phenomenon in about 1 in 6 million cases (Lancaster et al., 2010; Onu et al., 2013). CMT: Charcot-Marie-Tooth, HNPP: Hereditary neuropathy with liability to pressure palsies, MPZ: Myelin protein zero, ND: No data, *NF1*: Neurofibromin 1, *NF2*: Moesin-Ezrin-Radixin Like (MERLIN) Tumor Suppressor, *PMP22*: Peripheral myelin protein 22.
