# Supplementary material for: Nerve Enlargement in Patients with INF2 Variants Causing Peripheral Neuropathy and Focal Segmental Glomerulosclerosis
Source: Biomedicines. 2025 Jan 8;13(1):127. doi: 10.3390/biomedicines13010127 (PMC11763285; doi:10.3390/biomedicines13010127)
Supplement: Supplementary file 1 [file biomedicines-13-00127-s001.zip › R1 Ht Supplementary Tables S3.pdf]

**Supplementary Table S3. Case reports showing the nerve hypertrophy in CMT**

| | Referrences | CMT Neuropathy | | | Hypertrophic nerve changes | | Notes |
|---|---|---|---|---|---|---|---|
| | | Subtype | Mutations | Initinal manifestation | Age onset of years Gender | Affection locations | |
| **I** | **Spine and Extremities** | | | | | | |
| 1 | Morano J. U.,1986 [65] | CMT | N.D. | Weakness in feet and decreased sensation in distal extremities<br>Difficulty in movement of hands and fingers | 59, M | "Onion-bulb" formation<br>Nerve roots enlarged in cervical and lumbar regions | Thorasic regions not examined |
| 2 | Choi S.K., 1990 [66] | CMT | N.D. | Chronic back pain | 41, M (23)* | cauda equina, nerve roots at L3-S1, and ganglia. | |
| 3 | Bütefisch C., 1999 [21] | CMT1A | PMP22 duplication | Losing balance<br>Numbness<br>Weakness of left thigh<br>Clumsiness of right hand<br>Urination and defecation urgency | 44, M | Cervical C4-C7<br>Lumbar T12-S2<br>Cauda equina L2-L4 | demyelinating, NCV 18-25 m/sec upper limb |
| 4 | Pareyson D., 2003 [10] | CMT1A | PMP22 duplication<br>Copy number n=4 | Foot deformities, moderate scoliosis<br>Bilateral hearing loss<br>Muscle wasting and weakness<br>Generalized areflexia<br>Distal sensory loss | 45, M | Surral and Ulnar nerve<br>"Onion bulbs"<br>Cauda equina root | Rare PMP22 tetrasomy<br>High CSF protein |
| 5 | Liao J. P., 2004 [67] | CMT1A | PMP22 duplication | Pes cavus deformities | 21, M | Lumbar nerve roots L4-5, cervical | |
| 6 | Wadhwa V, 2012 [68] | CMT1A | PMP22 duplication | | 27, F | Obturator nerves, sciatic plexuses, and Femoal nerve | |
| 7 | An H., 2017 [69] | CMT1A | PMP22 duplication | Lower extremeties weakness | 28, F | cauda equina<br>nerve roots (C5-C7, L3-S3)<br>"onion bulb" | Sural nerve Biopsy loss of large myelinated fiber |
| 8 | Shibuya K., 2019 [70] | CMT1A | PMP22 duplication | Sensory disturbance and feet weakness | 35, M | median and ulnar nerves | demyelinating, NCV 11-28 m/sec upper limb |
| **II** | **Cranial nerves** | | | | | | |
| 1 | Aho T. R., 2004 [57] | CMT HNPP | PMP22 deletion | Lower extremeties weakness<br>Bilateral deafness<br>Facial neuralgia | 64, M | Cranial nerve VII<br>Cisternal segments of cranial nerve III, V (V3, V2 segments) | |

**Abbreviations**
CMT: Charcot-Marie-Tooth disease
HNPP: Hereditary neuropathy with liability to pressure palsies
NF1: Neurofibromatosis 1
GJB1: gap junction protein beta1; Cx32
MFN2: Mitofusin-2
MPZ: Myelin protein zero
NEFL: Neurofilament light polypeptide
EGR2: Early growth response protein 2
ARHGEF10: Rho guanine nucleotide exchange factor 10
PMP22: peripheral myelin protein 22
