# Supplementary material for: Nerve Enlargement in Patients with INF2 Variants Causing Peripheral Neuropathy and Focal Segmental Glomerulosclerosis
Source: Biomedicines. 2025 Jan 8;13(1):127. doi: 10.3390/biomedicines13010127 (PMC11763285; doi:10.3390/biomedicines13010127)
Supplement: Supplementary file 1 [file biomedicines-13-00127-s001.zip › R1 Ht Supplementary Tables S4.pdf]

Supplementary Table S4. Comparison of peripheral nerve enlargement among CMT and other demyelinating disorders

	Referrences	CMT Neuropathy						Hypertrophic nerve changes	Notes
		Subtype	n	Mutations	Age onset(Years)	Gender Ratio (M/F)	Modality		
	**Comparison CMT1A with other subtypes**								
1	Padua L, 2018 [70]	CMT1	30	electrophysiologocally demyelinating	45.5	0.9	US		
		CMT1A	17	PMP22 dup	46	1.1	upper limb	Nerve CSA were larger in CMT1 than in other subtypes. CSAs of the median and ulnar nerves were much larger at the proximal site	
		CMT2	22	MFN2, Rab7, TRPV4, NEFL, MPZ , GDAP1	61.5	0.7			
		CMTX	8	GJB1	42	0.6			
		NHNPP	10	PMP22 deletion	51.5	0.7			
2	Noto YI, 2015 [72]	CMT1A	20	PMP22 duplication	47.6	1.0	US		
		MPZ-associated CMT	3	MPZ	39.7	2	median, sural	CSA of all nerves(median, sural, great auricular, C6 nerve roots) were significantly increased in CMT1A than in control (n=30)	
		NEFL-associated CMT	4	NEFL	47.3	1	auricular, C6 root		
		EGR2-associated CMT	1	EGR2	49.0	1 (F only)			
		ARHGEF10-associated CMT	1	ARHGEF10	67.0	1 (M only)			
3	Schreiber S, 2013 [73]	CMT1A	12	PMP22 dup	42 ± 16.6 (13-72)	0.81	US	Median nerve CSA was significantly increased in CMT1A and correlated with NCV slowing	
		CMT2A	7	MFN2			Median nerve		
		CMTX	5	GJB1					
		HNPP	5	PMP22 deletion					
4	Elmansy M, 2022 [74]	CMT1A	20		46.6±14.5		MRI	CSAs of Scitic and Tibial nerve were enlarged in CMT1A than in control	
		IBM	20	Inclusion body myositis	66.9±8.8		Lower limb		
5	Martinoli C, AJR 2001 [75]	CMT1A	12		39.9±13.4 (three subgroup)	1.4	US<br>Median nerve	CMT1A can be distingushied by a larger nerve CSA and fascicular diameter from other subtypes (CMT2, CMTX).	
		CMT2	7						
		CMTX	5						
6	Cellerini M, 2000 [35]	CMT1	5		35 to 43		MRI		
		CMT2	2		39,42		Cauda quina	Seven CMT cases showed abnormal MRI: interdural nerve root hypertrophy (n=2), T-2 brightness(n=2) and enhancement (n=3)	
		DSD	3		28, 63, 65				
7	Pazzaglia C., 2013 [76]	CMT1A	20		44.2 (16-62)	0.4	US<br>Ulnar and sural nerve	CSA in ulnar but not sural nerve was enlarged	
	**Comparison between CMT1A and CIDP/other neuropathy subtypes**								
1	Naito H, 2024 [77]	CMT	12	demyelinating	range 33-83	1.1	US Lower Extremity	Enlagement site numbers (ENSs) in the intermediate and lower extremities were greater in demyelinating CMT than in CIDP	
		CIDP	17		range 28-84)	3			
2	Grimm A, 2016 [53]	CMT1	13	PMP22 dup n=10, MPZ n=3	40(7-52)	2.25	US	Nerve CSA was significantly enlarged in all neuropathies. The enlargement was most prominent in CMT1 subgroup.	
		CIDP	27	a)	63(24-79)	8	ulnar, median		
		MADSAM	10	b)	68(48-80)	4	tibial		
		MMN	12	c)	49(28-70)	12			
3	Sugimoto T, 2013 [52]	CMT	10	demyelinating	range 23-84	2.3	US	NCAs were larger in nerve trunk, but not roots, at all examined sites in the CMT than in CIDP.	
		CIDP	16		range 30-85	2.2	median and ulnar nerves and the cervical nerve roots C5, C6		
4	Sinclair CDJ, 2011 [78]	CMT1A	10		42.5±13.7		MRI<br>sciatic nerve	Sciatic nerve CSA, enlarged in both CMT1A and CIPP: median area CMT1A 135.9>67.6 mm2 CIDP vs control 43.3 mm2	
		CIDP	9		47.0±13.4				
5	Zaidman, 2009 [79]	CMT1A	11		range 2 to 69				
		CIDP	36		range 4 to 81		US<br>Median and ulnar nerve cross-sectional areas	Frequency of nerve enlargement and nerve size index were greater in CMT1A than in CIDP. Nerves were enlarged in all CMT-1A, 86% CIDP, 47% GBS, 19% Axonal neuropathy	
		GBS	17		range 8 to 82				
		axonal neuropathy	36		range 19 to 84				
6	Ellegala DB,2005 [80]	CMT1A	3		34,65, 84		MRI		
		CMT1B	1		38				
		CMT2	1		32			MRI T2 brightness was higher in CMT1A, CMT1B and CMTX than in CMT2, HNPP and CIDP	
		CMTX	1		55				
		HNPP	1		51				
		CIDP	1		37				
7	Midroni G., 1999 [22]	CMT1A	5						
		CIDP	16		40(18-73)		MRI	Root enlargement in 3/16 CIDP (19%)<br>Root enlargement in 1/5 CMT1A (20%)	
		Motorneuronal disease	2						
		Diabetic polyradiculopathy	1						
		Spinocerebellar degeneration	1						
		Hypertrophic brachial plexus neuropathy	1						
		Spinal stenosis	1						
		Transverse myelitits	1						

Abbreviation

CIDP: Chronic inflammatory demyelinating polyradiculoneuropathy
DSD: Dejerine-Sottas syndrome
PMP22: peripheral myelin protein 22
MPZ: Myelin protein
GJB1: gap junction protein beta1 (Connexin 32)
MFN2: Mitofusin 2
IBM: Inclusion body myositis
GBS: Guillain-Barré syndrome
NEFL: Neurofilament light chain
EGR2: Early growth response gene-2
ARH GEF10: Rho Guanine Nucleotide Exchange factor 1
HNPP: Hereditary neuropathy with liability to pressure palsies
CSA: Cross sectional area
a) Chronic Inflammatory demyelinating polyradiculoneuropathy
b) Multifocal acquired demyelinating sensor and motor neuropathy
c) Multifocal motor neropathy
