# Supplementary material for: Nerve Enlargement in Patients with INF2 Variants Causing Peripheral Neuropathy and Focal Segmental Glomerulosclerosis
Source: Biomedicines. 2025 Jan 8;13(1):127. doi: 10.3390/biomedicines13010127 (PMC11763285; doi:10.3390/biomedicines13010127)
Supplement: Supplementary file 1 [file biomedicines-13-00127-s001.zip › R3 by Supplementary References Dec 31 2024.pdf]

Supplementary References

Nerve Enlargement in Patients with INF2 Variants Causing Peripheral Neuropathy and Focal Segmental Glomerulosclerosis by Quynh TTH, Linh TNT et al

Supplemenary Figure S5

62. Bradley, W. G., Disorders of peripheral nerves. Oxford, Blackwell Scientific Publications. **1974**.

63. Abbas, A. K.; Fausto, N.; Robbins, S. L., *Robbins and Cotran pathologic basis of disease*. Elsevier Saunders: 2005.

64. Yiu, E. M.; Brockley, C. R.; Lee, K. J.; Carroll, K.; De Valle, K.; Kennedy, R.; Rao, P.; Delatycki, M. B.; Ryan, M. M., Peripheral nerve ultrasound in pediatric Charcot-Marie-Tooth disease type 1A. *Neurology* **2015,** *84* (6), 569-574.

Supplementary Table S3

65. Morano, J. U.; Russell, W. F. J. R., Nerve root enlargement in Charcot-Marie-Tooth disease: CT appearance. *Radiology* **1986,** *161* (3), 784-784.

66. Choi, S. K.; Bowers, R. P.; Buckthal, P. E., MR imaging in hypertrophic neuropathy: a case of hereditary motor and sensory neuropathy, type I (Charcot-Marie-Tooth). *Clinical imaging* **1990,** *14* (3), 204-207.

67. Liao, J. P.; Waclawik, A. J., Nerve root hypertrophy in CMT type 1A. *Neurology* **2004,** *62* (5), 783-783.

68. Wadhwa, V.; Thakkar, R. S.; Maragakis, N.; Höke, A.; Sumner, C. J.; Lloyd, T. E.; Carrino, J. A.; Belzberg, A. J.; Chhabra, A., Sciatic nerve tumor and tumor-like lesions—uncommon pathologies. *Skeletal radiology* **2012,** *41*, 763-774.

69. An, H.; Li, J.; Wang, L.-M.; Cui, B.; Piao, Y.-S.; Ren, Y.-J.; Chen, H.; Wang, Y.-P.; Wu, L.-Y., A rarely concerned magnetic resonance image sign of spinal nerve root hypertrophy in type 1A Charcot-Marie-tooth disease. *Chinese Medical Journal* **2017,** *130* (22), 2767-2768.

70. Shibuya, K.; Yoshida, T.; Misawa, S.; Sekiguchi, Y.; Beppu, M.; Amino, H.; Suzuki, Y.-i.; Suichi, T.; Tsuneyama, A.; Nakamura, K., Hidden Charcot-Marie-Tooth 1A as revealed by peripheral nerve imaging. *Internal Medicine* **2019,** *58* (21), 3157-3161.

Supplementary Table S4

71. Padua, L.; Coraci, D.; Lucchetta, M.; Paolasso, I.; Pazzaglia, C.; Granata, G.; Cacciavillani, M.; Luigetti, M.; Manganelli, F.; Pisciotta, C., Different nerve ultrasound patterns in Charcot-Marie-Tooth types and hereditary neuropathy with liability to pressure palsies. *Muscle nerve* **2018,** *57* (1), E18-E23.

72. Noto, Y.-i.; Shiga, K.; Tsuji, Y.; Mizuta, I.; Higuchi, Y.; Hashiguchi, A.; Takashima, H.; Nakagawa, M.; Mizuno, T., Nerve ultrasound depicts peripheral nerve enlargement in patients with genetically distinct Charcot-Marie-Tooth disease. *J. Neurol. Neurosurg. Psychiatry* **2015,** *86* (4), 378-384.

73. Schreiber, S.; Oldag, A.; Kornblum, C.; Kollewe, K.; Kropf, S.; Schoenfeld, A.; Feistner, H.; Jakubiczka, S.; Kunz, W. S.; Scherlach, C., Sonography of the median nerve in CMT1A, CMT2A, CMTX, and HNPP. *Muscle nerve* **2013,** *47* (3), 385-395.

74. Elmansy, M.; Morrow, J. M.; Shah, S.; Fischmann, A.; Wastling, S.; Reilly, M. M.; Hanna, M. G.; Helmy, E. M.; El-Essawy, S. S.; Thornton, J. S., Evidence of nerve hypertrophy in patients with inclusion body myositis on lower limb MRI. *Muscle nerve* **2022,** *66* (6), 744-749.

75. Martinoli, C.; Schenone, A.; Bianchi, S.; Mandich, P.; Caponetto, C.; Abbruzzese, M.; Derchi, L. E., Sonography of the median nerve in Charcot-Marie-Tooth disease. *American Journal of Roentgenology* **2002,** *178* (6), 1553-1556.

76. Pazzaglia, C.; Minciotti, I.; Coraci, D.; Briani, C.; Padua, L., Ultrasound assessment of sural nerve in Charcot–Marie-Tooth 1A neuropathy. *Clin Neurophysiol* **2013,** *124* (8), 1695-1699.
77. Naito, H.; Sugimoto, T.; Hironaka, A.; Nakamori, M.; Yamazaki, Y.; Ochi, K.; Maruyama, H., Diagnostic value of lower extremity ultrasonographic nerve enlargement for differentiating demyelinating Charcot-Marie-Tooth disease from chronic inflammatory demyelinating polyneuropathy. *J Neurol Sci* **2024,** *460*, 122995.
78. Sinclair, C.; Miranda, M.; Cowley, P.; Morrow, J.; Davagnanam, I.; Mehta, H.; Hanna, M.; Koltzenburg, M.; Reilly, M.; Yousry, T., MRI shows increased sciatic nerve cross sectional area in inherited and inflammatory neuropathies. *J. Neurol. Neurosurg. Psychiatry* **2011,** *82* (11), 1283-1286.
79. Zaidman, C. M.; Al-Lozi, M.; Pestronk, A., Peripheral nerve size in normals and patients with polyneuropathy: an ultrasound study. *Muscle nerve* **2009,** *40* (6), 960-966.
80. Ellegala, D. B.; Monteith, S. J.; Haynor, D.; Bird, T. D.; Goodkin, R.; Kliot, M., Characterization of genetically defined types of Charcot-Marie-Tooth neuropathies by using magnetic resonance neurography. *J. Neurosurg* **2005,** *102* (2), 242-245.

Supplementary Table S5

81. Koc, F.; Guzel, A. I., Neurofibromatosis type 1 associated with Charcot–Marie–Tooth type 1A. *The Journal of Dermatology* **2009,** *36* (5), 306-311.
82. Lupski, J. R.; Pentao, L.; Williams, L. L.; Patel, P. I., Stable inheritance of the CMT1A DNA duplication in two patients with CMT1 and NF1. *Am J Med Genet* **1993,** *45* (1), 92-96.
83. Onu, D. O.; Hunn, A. W.; Peters-Willke, J., Charcot-Marie-Tooth syndrome and neurofibromatosis type 1 with multiple neurofibromas of the entire spinal nerve roots. *Case Reports* **2013,** *2013*, bcr2013010078.
84. Ma'luf, R. N.; Baha'N, N.; Ghazi, N. G.; Tawil, A. N.; Allam, S. S., Bilateral, localized orbital neurofibromas and Charcot-Marie-Tooth disease. *Archives of Ophthalmology* **2005,** *123* (10), 1443-1445.
85. Bosch, E. P.; Murphy, M. J.; Cancilla, P. A., Peripheral neurofibromatosis and peroneal muscular atrophy. *Neurology* **1981,** *31* (11), 1408-1408.
86. Roos, K.; Pascuzzi, R.; Dunn, D., Neurofibromatosis, Charcot-Marie-tooth disease, or both? *Neurofibromatosis* **1989,** *2* (4), 238-243.
